# Rigorous Process for Isolation of Gut-Derived Extracellular Vesicles (EVs) and the Effect on Latent HIV

**DOI:** 10.3390/cells14080568

**Published:** 2025-04-09

**Authors:** Nneoma C. J. Anyanwu, Lakmini S. Premadasa, Wasifa Naushad, Bryson C. Okeoma, Mahesh Mohan, Chioma M. Okeoma

**Affiliations:** 1 Department of Pathology, Microbiology, and Immunology, New York Medical College, Valhalla, NY 10595-1524, USA; wnaushad@nymc.edu (W.N.); 2 Host Pathogen Interaction Program, Southwest National Primate Research Center, Texas Biomedical Research Institute, San Antonio, TX 78227-5302, USA; 3 Lovelace Biomedical Institute, Albuquerque, NM 87108-5127, USA

**Keywords:** extracellular vesicles (EVs), extracellular condensates (ECs), GI-derived EVs, EVs|ECs, particle purification liquid chromatography (PPLC), polyvinylpolypyrrolidone (PVPP)

## Abstract

The human gastrointestinal (GI) track host trillions of microorganisms that secrete molecules, including extracellular vesicles (EVs) and extracellular condensates (ECs) that may affect physiological and patho-physiological activities in the host. However, efficient protocols for the isolation of pure and functional GI-derived EVs|ECs is lacking. Here, we describe the use of high-resolution particle purification liquid chromatography (PPLC) gradient-bead-column integrated with polyvinylpolypyrrolidone (PVPP)-mediated extraction of impurities to isolate EVs from colonic content (ColEVs). PVPP facilitates the isolation of pure, non-toxic, and functionally active ColEVs that were internalized by cells and functionally activate HIV LTR promoter. ColEVs isolated without PVPP have a reductive effect on MTT (3-(4,5-dimethylthiazol-2-yl)-2,5-diphenyltetrazolium bromide) without living cells, suggesting that ColEVs contain reductases capable of catalyzing the reduction of MTT to formazan. The assessment of the origin of ColEVs reveals that they are composed of both bacteria and host particles. This protocol requires ~12 h (5 h preprocessing, 7 h isolation) to complete and should be used to purify EVs from sources contaminated with microbial agents to improve rigor. This protocol provides a robust tool for researchers and clinicians investigating GI-derived EVs and the translational use of GI-derived EVs for diagnostic and therapeutic use. Additionally, GI-derived EVs may serve as a window into the pathogenesis of diseases.

## 1. Introduction

It is now clear that EVs encompass subgroups of exosomes, microvesicles, and other membranous vesicles, with overlapping size, density, surface charge, and markers [1]. ECs, on the other hand, comprise exomeres and supermeres that are cytosolic aggregates of lipids, proteins, and RNAs. Body fluids [2,3,4,5,6,7,8,9,10,11,12,13,14,15,16], brain [17], and gut [18] contain ECs|EVs. Cells from the three kingdoms of life––Eukarya, Bacteria, and Archaea—secrete extracellular particles (EPs), particularly EVs. Interestingly, ECs|EVs from one species may function in another species, and the interspecies efficacy of ECs|EVs and their cargo has been established by our group and others [17,19]. ECs|EVs act in a paracrine fashion as regulators of health and diseases, including infectious diseases, non-communicable diseases, drug abuse, injury, and reproduction, and they modulate cellular functions and/or dysfunctions [20,21,22]. In infectious diseases, ECs|EVs regulate viral replication and host response to infection [8,23,24,25,26] and regulate parasitic (chagas) disease [27,28,29,30]. In cancer research, EVs are explored as diagnostic biomarkers [31,32,33,34,35], drug discovery, delivery, and therapeutics [36,37,38,39,40,41]. Aside from infectious diseases and cancer, EVs play key roles in the study of the pathogenesis of Asthma [42,43], cardiovascular diseases [44], and arthritis [45,46,47], as well as in orthopedics and regenerative medicine [48,49,50]. The direct interaction between ECs|EVs and cells has become the basis of a short- or long-distance intercellular communication mechanism, whereby ECs|EVs trigger a cellular response in host/recipient cells. Most cells respond in tissue-specific ways to ECs|EVs cargo. Thus, ECs|EVs research has become a subject of interest to scientists and clinicians across numerous disciplines from basic sciences (chemistry and biology) to applied sciences (diagnostics and pharmaceutics) to medicine. While ECs|EVs isolated from biofluids and tissues have extensively been studied, less attention has been paid to GI-derived ECs|EVs, despite the involvement and importance of the gut microbiome in health and disease, especially in the area of microbiota–gut–brain axis research. Such focus in the ECs|EVs field prompted our interest in understanding the interkingdom roles of ECs|EVs, with a focus on GI-derived ECs|EVs. However, there is a paucity of information on the effective isolation technique for GI-derived ECs|EVs.

The purity of EVs|ECs, which ultimately depends on the isolation method used to obtain them is critical, particularly EVs|ECs from the GI tract that is colonized by a huge number of enteric (pathogenic and commensal) bacteria [51]. Therefore, identifying GI derived EVs|ECs of bacterial and host origin may serve as a window into the pathogenesis of diseases and as a potential therapeutic target.

Here, we demonstrate rigorous protocol that includes pre-treatment with 1% polyvinylpolypyrrolidone (PVPP) for isolating highly purified EVs from colonic contents (ColEVs) using PPLC [16,52,53,54]. PVPP is a synthetic polymer of N-vinylpyrrolidone that is insoluble in water, alcohol, and acid [55]. PVPP is widely used in the food and beverage industry as a fining agent for the removal of phenolic compounds (flavonoids and non-flavonoids) [56] via noncovalent hydrogen bonding between phenol groups and CO─N linkages in the structure of PVPP [57]. The addition of PVPP in brewing helps to prevent haze formation, reduce bitterness and astringency, as well as improve color stability [58]. Although PVPP has affinity for phenolic compounds, it has poor selectivity and as such, removes resveratrol from wine [59], which negatively affects the health benefits of wine.

Our data revealed that the pre-treatment of colonic contents with 1% PVPP improved the quality of isolated ColEVs as assessed by the responses of the target cells to ColEVs isolated with and without PVPP.

## 2. Materials and Methods

### 2.1. Experimental Design, Animal Care, and Institutional/Ethical Approvals

All experiments using rhesus macaques were approved by the Tulane and LSUHSC Institutional Animal Care and Use Committee (IACUC, Protocols 3574, 3581, and 3781) that was approved on 16 August 2023, and does not expire until 16 August 2026. The Tulane National Primate Research Center (TNPRC, Covington, LA, USA) is an association for Assessment and Accreditation of Laboratory Animal Care International-accredited facility (AAALAC #000594). The NIH Office of Laboratory Animal Welfare assurance number for the TNPRC is A3071-01. All clinical procedures, including administration of anesthesia and analgesics, were carried out under the direction of a laboratory animal veterinarian. Animals were pre-anesthetized with ketamine hydrochloride, acepromazine, and glycopyrrolate, intubated and maintained on a mixture of isoflurane and oxygen. All possible measures were taken to minimize the discomfort of all the animals used in this study. Tulane University complies with NIH policy on animal welfare, the Animal Welfare Act, and all other applicable federal, state and local laws.

### 2.2. Biospecimens

Colon content specimens used for this study were obtained from weight-matched specific-pathogen-free (free of D retrovirus, Herpes B SIV, and STLV), opportunistic infection and antibiotic free male Indian *Rhesus macaques* (RM, Mamu-A0*1^−^/B08^−^/B17^−^). Colon surgical resection segments (~5 cm circular piece of intact colon) were obtained using our standard operating protocol. The segments were split open using sterile scissors and contents collected into 50 mL tubes containing RPMI media, centrifuged and stored frozen as 1.5 aliquots in cryovials. Samples were thawed and used for EV extraction.

### 2.3. Cell Lines

The following reagents were obtained through the NIH HIV Reagent Program that has transitioned into the Biological and Emerging Infections Research Resources Program (BEI-RRP), NIAID’s centralized research reagent repository known as BEI Resources at www.beiresources.org (accessed on 17 October 2022): TZM-GFP Human Cell Line (JC.53 Derived), HRP-20041, contributed by David G. Russell and David W. Gludish [60]; J-Lat Tat-GFP (ARP-9851) cells, contributed by Dr. Eric Verdin [61,62]. The J-Lat Tat-GFP and TZM-GFP cells were maintained in RPMI and DMEM media, respectively, containing 5% EV-depleted FBS (Gibco, Thermo Fisher Scientific, Waltham, MA, USA), 100 U/mL penicillin, 100 μg/mL streptomycin and sodium pyruvate, and 0.3 mg/mL L-glutamine (Invitrogen, Molecular Probes, Thermo Fisher Scientific, Waltham, MA, USA) as previously described [8].

### 2.4. Preprocessing of Colonic Contents (Thawing and Pre-Treatment with PVPP) and Isolation of ColEVs

All colon content samples were stored at −80 °C prior to processing. A starting volume of between 1 and 2 mL was thawed at room temperature in the biosafety cabinet. All thawed samples were processed before storing for isolation, if isolation is not possible on the same day. The method used for thawing and separation of the colonic content is provided in Supplementary Materials. If isolation is not possible on the same day, samples were stored at −80 °C. Prior to the isolation of ColEVs, the 100 cm gradient PPLC column was packed with multi-sized beads as previously described [53]. The ColEVs were isolated with 0.1X PBS and stored as previously described [53]. Details can be viewed in Supplementary Materials.

### 2.5. Nano Tracking Analysis (NTA)

Samples were diluted to the appropriate concentration in filtered 0.1 X PBS for NTA. Size distribution, particle concentration and Zeta potential (ζ-potential) of the purified ColEVs were determined using ZetaView PMX110 (Particle Metrix, Mebane, NC, USA). First, the ZetaView machine and software were initialized, followed by a CellCheck to ensure the measurement cell. The cell assembly was flushed with deionized water to remove any contaminants. An alignment suspension (dilution 1:250,000) was then used to calibrate the instrument, which involved injecting the suspension, avoiding air bubbles, and allowing the instrument to perform auto-alignment and focus optimization. The system was calibrated using 100 nm Nanosphere™ size standards (3100A, Thermo Fisher Scientific, Waltham, MA, USA) before acquisition of measurements. The instrument parameters were set up according to recommendations [63]. Samples were then introduced into the chamber (in sample 1:1000 0.1X DPBS dilutions) and measurements were taken in triplicates at multiple positions to ensure accuracy. The ZetaView tracked the Brownian motion of particles to determine their size and concentration, utilizing sliding optics to move between focal positions and sample a large volume of the solution. For size, automated analysis of the 11 positions was performed, any outlier position was removed, and the median number (X50) was used to report the particle size. The measured concentration was normalized to the original volume of ColEVs before dilution and reported in particles/mL. Additionally, the instrument measured the zeta potential of particles by generating particle drift in an electrical field.

### 2.6. Labeling of ColEVs and Uptake of EVs by TZM-GFP and JLAT-TAT-GFP Cells

We determined EV uptake by TZM-GFP and JLAT-TAT-GFP cells at various incubation periods. Then, 1 µL DiR (5 µM DiR- 1,1-dioctadecyl-3,3,3,3-tetramethy-lindotricarbocyanine iodide—in 0.1X PBS) was added to 100 µg of EV and incubated at 37 °C for 30 min in the dark (inverted every 5 min). The ColEVs were quantified using Bradford assay. The mix was purified in EX03-8, Exo-spin columns mini (CellGS LLC, St. Louis, MO, USA) by first, washing column with 180 µL of 0.1X PBS and running 100 µL labeled EV through the column. Then, 180 µL 0.1X PBS was then run through column, and the purified EV was eluted with 50 µL 0.1X PBS. Concentrations of labeled EV used for uptake were 0 (PBS control), 50, 100, 150 and 200 µg each in 10,000 TZM-GFP or JLAT-TAT-GFP cells. For the JLAT-TAT-GFP cells, at 24 h incubation, 1 drop per 5 mL of NucBlue working solution was added, 100 µL per well, and incubated at room temperature for 5 min before imaging. Uptake was captured and analyzed at Brightfield, DAPI, GFP and Texas Red channels.

### 2.7. Western Blot

We also determined the presence of EV-specific tetraspanin proteins—CD63 and CD9—by Western blotting analysis. Protein concentration of the EVs were normalized via Bradford assay using Quick Start™ Bradford BSA standards (cat #5000201, Bio-Rad Laboratories, Inc., Hercules, CA, USA). Then, 20 µg analytes representing ColEVs or ColECs were boiled at 95 °C in the presence of 4X Laemmli buffer (cat #1610747, Bio-Rad Laboratories, Inc., Hercules, CA, USA) for 5 min before loading onto a 4–15% Tris-glycine gradient gel. The proteins were transferred onto a PVDF membrane overnight at 35 V constant volt in a cold room. The membrane was then blocked with 5% BSA for 1 h before incubating overnight (at 4 °C) with primary antibodies (1:1000) against CD63, CD9 (mouse, Developmental Studies Hybridoma Bank, DSHB, Iowa City, IA, USA). After 3 washes, the blots were incubated for 2 h at room temperature with fluorescent-labeled secondary IRDye 800CW donkey anti-mouse IgG antibodies (LI-COR, Lincoln, NE, USA), the membranes were imaged with the LI-COR Odyssey Infrared Imaging System.

### 2.8. Transmission Electron Microscopy (TEM)

Formvar and carbon-coated 400-mesh copper grids (Electron Microscopy Sciences, Hatfield, PA, USA) were glow-discharged for 30 s using a PELCO easiGlow™ unit (Ted Pella, Inc., Redding, CA, USA) to render the surface hydrophilic. The sample solution was prepared by dispersing the specimen in an appropriate buffer to achieve a final concentration suitable for transmission electron microscopy (TEM) analysis. A 3–5 µL aliquot of the sample solution was applied to the glow-discharged grid and allowed to settle for 1 min at room temperature. Excess sample was removed by gently touching the edge of the grid with #1 filter paper (Whatman, GE Healthcare, Chicago, IL, USA) to wick away the solution by capillary action. The grid, with the sample side facing down, was immediately placed onto a 50 µL drop of 1.5% aqueous uranyl acetate (Electron Microscopy Sciences, Hatfield, PA, USA) on a sheet of parafilm (Bemis Company, Inc., Neenah, WI, USA). After 1 min of staining, the uranyl acetate was wicked off using filter paper, and the grid allowed to air dry for 5 min at room temperature. Samples were then viewed using a JEM-1400 transmission electron microscope (JEOL, Inc., Peabody, MA, USA) operated at 100 kV. Images were captured with a Veleta 2K × 2K CCD camera (EMSIS GmbH, Münster, Germany).

### 2.9. Activation of HIV LTR Promoter

We assessed the activation of HIV LTR promoter by running a 24 h kinetics on Lionheart FX Automated Microscope (SN: 18022029, Agilent Technologies, Santa Clara, CA, USA). Ten thousand (10,000) TZM-GFP cells each were seeded into four wells of a 24-well Costar^®^ 24-well Clear TC-treated multiple well plate (Cat. # 3524, Corning, NY, USA) and incubated for 3 h at 37 °C, 5% CO_2_ to allow for cell attachment. DiR-labeled EVs were prepared by adding 50 µg (per treatment/sample) of EVs to DiR in 0.1X PBS (1,1-dioctadecyl-3,3,3,3-tetramethylindotricarbocyanine) to reach a 5 µM final concentration. The labeled EVs were then passed through Cell Guidance EX03-8, Exo-spin columns mini (Cat. # EX03-8, Cell Guidance Systems LLC, St. Louis, MO, USA) for purification, and eluted with 0.1X PBS. Each well containing TZM-GFP cells was uniformly treated with purified EVs (50 µg). Then, 10 µL of NucBlue Live ReadyProbes Reagent (Catalog # R37606, Fisher Scientific, Waltham, MA, USA) was also added to each well just before placing in the Lionheart FX Automated Microscope (SN: 18022029, Agilent Technologies, Santa Clara, CA, USA).

Imaging was performed every hour in two batches, first for the first 13 h, then for the last 5 h (20th to 24th hour) using Gen5 3.15 software, configured for 10X magnification. The instrument temperature was set to 37 °C, and the CO_2_ pump was turned on for consistent 5% CO_2_ inflow. The kinetics option was selected on the Gen5 software, the imaging channels were set up for GFP (Excitation—488 nm, Emission—509 nm), DiR (Texas Red, Excitation—748 nm, Emission—780 nm), and NucBlue (DAPI, Excitation—360 nm, Emission—460 nm).

Images were acquired at multiple time points to monitor the kinetics of uptake of ColEVs. For each time point, images were captured in all four channels (GFP, Texas Red, DAPI, and Brightfield) for each well. The acquired images were processed using Gen5 3.15 software, with background subtraction using the image processing option.

The processed images were analyzed to quantify the uptake of ColEVs by measuring the fluorescence intensity of DiR-labeled EVs within GFP-positive cells. NucBlue staining was used to identify and analyze the nuclei, ensuring accurate cell counting. Using GraphPad Prism 10.3.1 (509), the normalized fluorescence intensity of the cells for each channel was plotted against time to generate a kinetic uptake curve. Data were normalized to the number of cells to account for variations in cell number across wells.

### 2.10. Sorting Colonic EVs Using Flow Cytometry

ColEVs were sorted with SONY benchtop SH800S Cell Sorter into ColEVs of bacterial and human origin. Two fluorescent PolyAn Nanobeads with Streptavidin-functionality were used for Sandwich incubation of ColEVs samples with 2 biotinylated antibodies—recombinant Escherichia coli multi-drug efflux pump subunit AcrA (acrA) and human CD9, which is a known marker of EVs. All preparations were made in a single tube (multiplex), and samples were separately sorted into two tubes: one tube for AcrA+ ColEVs and the other for CD9+ ColEVs.

### 2.11. Normalization Approach for Cell-Based Assays

We divided uptake or LTR values with NucBlue values for each image, and then multiplied by the initial cell number (10,000) and added to a pseudo-count (1).

### 2.12. Statistical Analysis

Significance cutoff was set to *p*-value <0.05. Statistical differences were assessed by one- or two- way ANOVA with Šídák’s or Tukey’s multiple comparisons test, two-stage linear step-up procedure of Benjamini, Krieger and Yekutieli, or Binary Student’s *t* tests (Welch’s correction) using GraphPad Prism 10. Details of specific statistics are on figure legends.

Details on modifications made to PPLC, packing and equilibration of PPLC column, the key steps involved in isolation of colonic ECs|EVs, general laboratory reagents and equipment can be found in the Supplementary Materials—step-by-step protocol.

## 3. Results

### 3.1. Development and Overview of the Protocol

**Figure 1:**
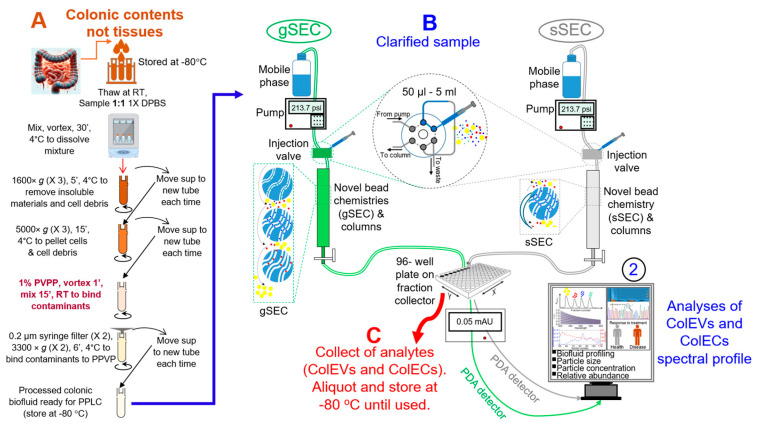
Schematic of PPLC workflow. (**A**) A total of 1–2 mL of colonic contents was pre-cleared through flash agitation, pre-incubation with 1% PVPP, rotation, spinning at varying temperatures, and filtration with 0.2 µm syringe filter. (**B**) The clarified samples were directly loaded onto gradient bead PPLC column (gSEC) or single bead PPLC column (sSEC) for ColEVs isolation. Shown in Processes gSEC, sSEC, 2 are the different aspects of PPLC all in one isolation, analysis, and retrieval steps. (**C**) This process is independent of PPLC but represents what can be done with the analytes. This protocol is adapted from [53] and modified for ColEVs. PVPP = Polyvinylpolypyrrolidone, gSEC = gradient bead size exclusion chromatography, sSEC = single bead size exclusion chromatography.

EPs are important research, diagnostic, therapeutic, and drug delivery tools. However, despite these great attributes, challenges abound on how to isolate EPs. Currently, there are several EP isolation methods, including ultracentrifugation, density gradient, ultrafiltration, flow cytometry, immunocapture, ion exchange or size exclusion chromatography (SEC), microfluidic, asymmetric flow field flow fractionation (AF4), and precipitation [54,64]. Each of these methods has specific limitations. Some of these methods may alter the properties of Eps, require special skills and expensive equipment, and copurify all analytes with other impurities [64,65]. The hope for separating analytes, such as ECs|EVs from impurities, relies on the ability to isolate and retrieve pure and functional ECs|EVs separately. This need inspired us to optimize PPLC [53,54], which employs an innovative gradient SEC (gSEC) protocol that facilitates high-resolution separation [53] for isolation of GI-derived EPs. Thus, in this protocol, we describe in detail the process of isolating bioactive and non-toxic ECs|EVs from colonic content of rhesus macaques. We also describe how to improve the purity of GI-derived ECs|EVs by the addition of impurity-adsorbent step using 1% PVPP [66]. This protocol for the isolation of gut-derived EVs is modified from our published protocols where we included preprocessing sequential centrifugations and treatment with PVPP (Figure 1A) to the published PPLC isolation [15,16,53,54] with fraction collection and spectral analysis with spectrophotometer (Figure 1B), as well as collection of different populations of analytes (ECs|EVs), aliquoting, and storage (Figure 1C). In previous studies, we described the use of PPLC to isolate, separate, and collect preparatory quantities of ECs and EVs from blood, semen, and tissues [15,16,53,54] but not from intestinal contents or the gut. Modifications made to PPLC and the key steps involved in isolation of colonic ECs|EVs can be found in the Supplementary Materials.

### 3.2. EVs but Not ECs Are Present in Colonic Contents of Rhesus Macaques

**Figure 2:**
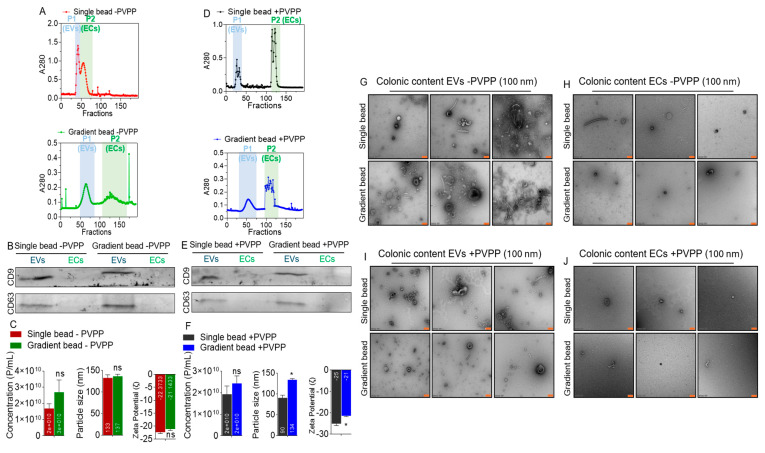
Integration of PVPP and PPLC bead column chemistry facilitates isolation of clean ColEVs: (**A**) Spectra of the analytes isolated with single or gradient beads in the absence of PVPP (-PVPP). (**B**) Western blot of known EV markers present in ColEVs isolated with single or gradient beads in the absence of PVPP. (**C**) Physical properties of ColEVs isolated with single or gradient beads in the absence of PVPP. (**D**) Spectra of the analytes isolated with single or gradient beads in the presence of PVPP (+PVPP). (**E**) Western blot of known EV markers present in ColEVs isolated with single or gradient beads in the presence of PVPP. (**F**) Physical properties of ColEVs isolated with single or gradient beads in the presence of PVPP. (**G**–**J**) TEM images (at 100 nm scale) of ColEVs isolated with single or gradient beads in the absence of PVPP (**G**,**H**) and in the presence of PVPP (**I**,**J**). P1 = Peak 1, P2 = Peak 2. Statistics were conducted with Prism Graph pad using Binary Student’s *t*-tests (Welch’s correction), * *p* < 0.05, and ns = non-significant. Scale bars = 100 nm.

Clarified supernatants from colonic contents obtained from RMs before infection and/or treatment (pretreatment), treated with or without PVPP were loaded atop (1) single-bead or (2) gradient-bead PPLC beds [53] to (i) gain insight into the spectra of colonic contents obtained through the different methods and (ii) identify and collect pure ColEVs devoid of other factors, such as ECs that often times co-purify with EVs [53]. Schematics for ColEVs isolation workflow are shown in Figure 1. The elution profiles of colonic digests from single bead without PVPP (-PVPP) is different from profile of gradient bead -PVPP (Figure 2A). The peaks labeled P1 for ColEVs and P2 for ColECs were collected and used for subsequent studies. Western blot assay for immunodetection of known markers of EVs showed that the isolated ColEVs were positive for the typical EV markers CD9 and CD63 while the P2 peak was negative (Figure 2B). The absence of the markers of EVs on the P2 (ECs) fraction is an indication that analytes in P2 may not be EVs. There were no differences in physical characteristics of ColEVs between single bead -PVPP and gradient bead -PVPP isolated ColEVs with regard to particle concentration (~1.7 × 10^10^ particles/mL vs. ~2.7 × 10^10^ particles/mL), size (~132.7 nm vs. 136.6 nm), and ζ-potential (−22.4 mV vs. −21.1 mV), respectively, for single-bead -PVPP and gradient-bead -PVPP ColEVs (Figure 2C).

In the presence of PVPP (+PVPP), the elution profiles of colonic digests from are different (Figure 2D). The peaks labeled P1 for ColEVs and P2 for ColECs were collected and used for subsequent analyses. The use of PVPP did not affect the presence of CD9 and CD63 because both markers were present in P1 (ColEVs), while the P2 peak was negative (Figure 2E). The particle concentration (~1.9 × 10^10^ particles/mL vs. ~2.4 × 10^10^ particles/mL) of single-bead +PVPP and gradient-bead +PVPP isolated ColEVs, respectively, were not different (Figure 2F, left). However, significant differences exist between single-bead +PVPP and gradient-bead +PVPP isolated ColEVs with regard to particle size (~90.3 nm vs. 133.5 nm, *p* = 0.0106: Welch’s correction), and ζ-potential (−22.4 mV vs. −21.1 mV, *p* = 0.0363: Welch’s correction), respectively, for single-bead +PVPP and gradient-bead +PVPP ColEVs (Figure 2F).

TEM analysis showed that EPs isolated with single and gradient bead columns without PVPP (-PVPP) contain ColEVs (Figure 2G) but not significant numbers of ColECs (Figure 2H). Similarly, single and gradient bead columns with PVPP (+PVPP) contain ColEVs (Figure 2I) but not significant numbers of ColECs (Figure 2J). The ColEVs are heterogenous in size, shape, and electron density irrespective of the use of PVPP or not. This said, ColEVs isolated with gradient-bead +PVPP (Figure 2I, bottom) seem cleaner compared to other methods. This TEM analysis confirmed that colonic content contains EVs but not ECs and agrees with the Western blot data presented in Figure 2B,E. While ECs are not enriched in colonic content, the data presented in Figure 2C,F show that the P2 analytes in Figure 2A,D have size, concentration, and ζ-potential data. This is not surprising because these may represent other particles in the extracellular space besides ColEVs and ColECs.

### 3.3. PVPP Facilitates Isolation of Pure ColEVs That Are Internalized by Human T Cells

**Figure 3:**
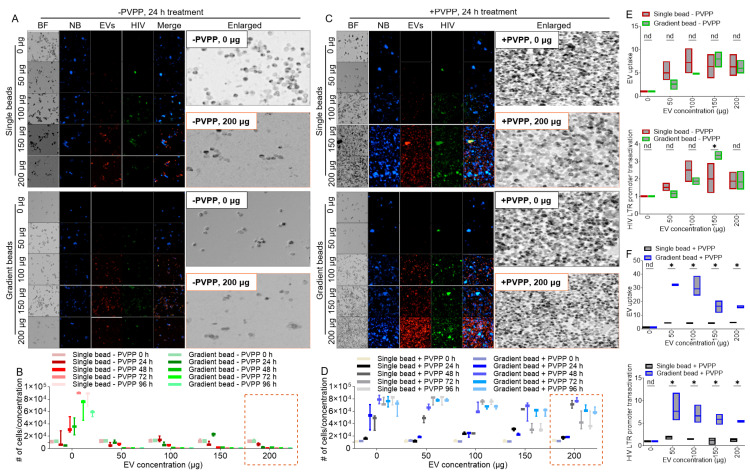
ColEVs are internalized by HIV latently infected JLAT-GFP T cells within 24 h and the ColEVs transactivated HIV LTR promoter while the clarifying PVPP promotes survival of cells treated with ColEVs. (**A**) Microscopic images of JLAT T cells treated with ColEVs isolated with single or gradient beads in the absence of PVPP. (**B**) Quantification of JLAT T cells treated with ColEVs isolated with single or gradient beads in the absence of PVPP. (**C**) Microscopic images of JLAT T cells treated with ColEVs isolated with single or gradient beads in the presence of PVPP. (**D**) Quantification of JLAT T cells treated with ColEVs isolated with single or gradient beads in the presence of PVPP. (**E**) Quantification of uptake (top) and level of HIV LTR promoter transactivation (bottom) of ColEVs isolated with single or gradient beads in the absence of PVPP and added to JLAT T cells. (**F**) Quantification of uptake (top) and level of HIV LTR promoter transactivation (bottom) of ColEVs isolated with single or gradient beads in the presence of PVPP and added to JLAT T cells. Statistics were conducted with Prism Graph pad using two-way ANOVA and two-stage linear step-up procedure of Benjamini, Krieger and Yekutieli. nd = no difference, *p* * ≤ 0.0001, ns ≥ 0.9999. BF = bright field, NB = NucBlue.

Internalization assays revealed that ColEVs labeled with lipophilic fluorescent membrane DiR deep red stain were taken up by JLAT-GFP T cells irrespective of whether they were isolated without PVPP (-PVPP, Figure 3A,B) or with PVPP (+PVPP, Figure 3C,D). Visual assessment of the images showed reduced DAPI signal in cells treated with ColEVs isolated with single- and gradient-bead -PVPP (Figure 3A) compared to those treated with ColEVs isolated with single- and gradient-bead +PVPP (Figure 3C). This observation indicates that ColEVs isolated without PVPP treatment is toxic to cells. To confirm this observation, we counted the number of cells in wells treated with different concentrations of ColEVs isolated with single- or gradient-bead -PVPP (Figure 3B) compared to ColEVs isolated with single- or gradient-bead +PVPP (Figure 3D). We confirmed that, in the absence of PVPP, ColEVs may be toxic to cells because the number of JLAT-GFP T cells significantly reduced from the starting seeding density of 10,000 cells (Figure 3B). In contrast, JLAT-GFP T cells treated with ColEVs isolated with single- or gradient-bead +PVPP significantly double from the starting seeding density of 10,000 cells to a mean of 20,214 vs. 26,905 at 24 h; 50,168 vs. 70,010 at 48 h; 60,955 vs. 68,789 at 72 h; 61,699 vs. 66,385 at 96 h, respectively, for single-bead +PVPP vs. gradient-bead +PVPP (Figure 3D). These data reveal that the addition of PVPP treatment in ColEVs isolation protocol may be a necessary step in preserving target cell viability.

Assessment of the uptake of ColEVs by cells revealed no difference in the uptake of single- or gradient-bead -PVPP isolated ColEVs (Figure 3E, top). However, in the presence of PVPP, internalization of ColEVs isolated with gradient beads was significantly higher compared to single-bead-isolated ColEVs (Figure 3F, top).

### 3.4. ColEVs Activate HIV LTR Promoter

Internalization of ColEVs is suggestive of possible bioactivity. In this regard, single- or gradient-bead -PVPP isolated ColEVs did not have a significant effect on transactivating HIV LTR promoter (Figure 3E, bottom). In comparison, gradient-bead +PVPP isolated ColEVs significantly increased HIV LTR promoter transactivation (Figure 3F, bottom). Both uptake of ColEVs and transactivation of HIV LTR promoter were assessed per cell. 50 µg of gradient-bead +PVPP had the highest effect on transactivating HIV LTR promoter (Figure 3F, bottom).

### 3.5. ColEVs Isolated in the Absence of PVPP Interfere with MTT Tetrazolium Reduction

**Figure 4:**
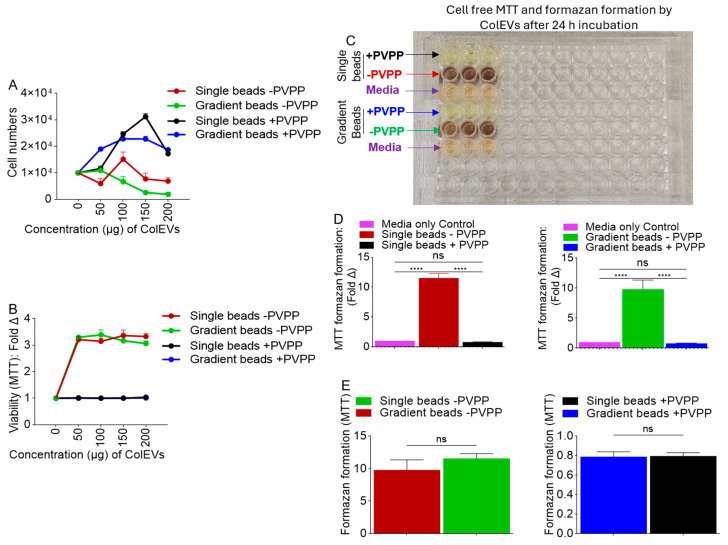
Effect of ColEVs on MTT reduction assay. (**A**) The effect of ColEVs on cell number is dependent on PVPP and not on bead:column chemistry. (**B**) The effect of ColEVs on cell viability is dependent on PVPP and not on bead:column chemistry. Statistics for panels A and B are presented in Supplementary Table S1 and Table S2, respectively. (**C**) ColEVs isolated in the absence of PVPP irrespective of bead:column type caused very strong increase in formazan formation resulting in colorimetric changes in MTT reduction compared to ColEVs isolated in the presence of PVPP irrespective of bead:column type. Red and green arrows = wells with ColEVs -PVPP; black and blue arrows = wells with ColEVs +PVPP; black rectangles = wells with media alone. (**D**,**E**) Quantification of MTT reduction by ColEVs isolated with single or gradient beads in the presence or absence of PVPP. Statistics were conducted with Prism GraphPad using unpaired *t* test with Welch’s correction. ns = not significant, **** *p* ≤ 0.0001.

Visual assessment of JLAT-GFP T cells treated with ColEVs, and enumeration of cell numbers (NucBlue staining and Brightfield) showed that ColEVs isolated in the absence of PVPP (-PVPP), irrespective of column type (single vs. gradient bead) significantly decreased cell numbers (Figure 3A). In contrast, ColEVs isolated in the presence of PVPP (+PVPP) had no effect on cell numbers (Figure 3B), suggesting that in the absence of PVPP, ColEVs may have toxic effects on cells. Given the observations on cell numbers in Figure 3, we tested the effects of ColEVs on cell numbers (NucBlue staining) and cell viability using the MTT tetrazolium assay. While cell numbers decreased for wells seeded with cells in the presence of 100 µg of ColEVs isolated without PVPP (-PVPP, Figure 4A, Supplementary Table S1), MTT values increased in a concentration independent manner (Figure 4B, Supplementary Table S2). In contrast, cells treated with 100 µg of ColEVs isolated with PVPP increased in number (Figure 4A, Supplementary Table S1), while MTT values did not change and was lower compared to the values from cells treated with ColEVs isolated without PVPP (+PVPP, Figure 4B, Supplementary Table S2). These data suggest a direct interaction of ColEVs -PVPP with the MTT tetrazolium reduction.

### 3.6. Direct Reductive Potential of MTT Absorbance by ColEVs -PVPP in a Cell-Free System

To confirm direct reductive potential of ColEVs, we used a cell-free system to assess the effects of ColEVs -PVPP and ColEVs +PVPP on tetrazolium reduction. Thus, ColEVs isolated with PVPP (+PVPP) or without PVPP (-PVPP) and with single or gradient columns were incubated with MTT and the absorbance was measured at 595 nm. Since color formation serves as a useful and convenient marker in the MTT assay, the results showed that wells containing ColEVs -PVPP had intense color change irrespective of whether the ColEVs were isolated with single vs. gradient beads (Figure 4C, red and green arrows). In contrast, ColEVs +PVPP isolated with single and gradient did not change color irrespective of the of whether the ColEVs were isolated with single vs. gradient beads (Figure 4C, black and blue arrows). The colors in wells containing ColEVs +PVPP (Figure 4C, black and blue arrows) were similar to the colors of the wells containing media alone (Figure 4C, purple arrows). The maximal absorbance values were significantly different, which were 0.79 (single bead, +PVPP) vs. 11.5 (single bead, -PVPP) as shown in Figure 4D, left; and 0.79 (gradient bead, +PVPP) vs. 9.79 (gradient bead, -PVPP), as shown in Figure 4D, right. The reductive potential of MTT absorbance by ColEVs is dependent on the absence (Figure 4E, left) or presence (Figure 4E, right) of PVPP and not the PPLC column because MTT absorbance did not significantly change based on whether single or gradient bead column was used (Figure 4E). These results support interference in MTT data published by others [67,68] and suggest that without the addition of PVPP, ColEVs reduce MTT in the absence of living cells. Based on these data, the isolation method used for ColEVs can significantly influence the results of experiments using the MTT assay to measure the effects of EVs on cell growth. Subsequent studies will be conducted with ColEVs +PVPP isolated with single vs. gradient beads to identify which isolation method is superior with regard to the bioactivity of ColEVs.

### 3.7. ColEVs Isolated with Single Beads Are More Effective in Transactivating HIV LTR Promoter

**Figure 5:**
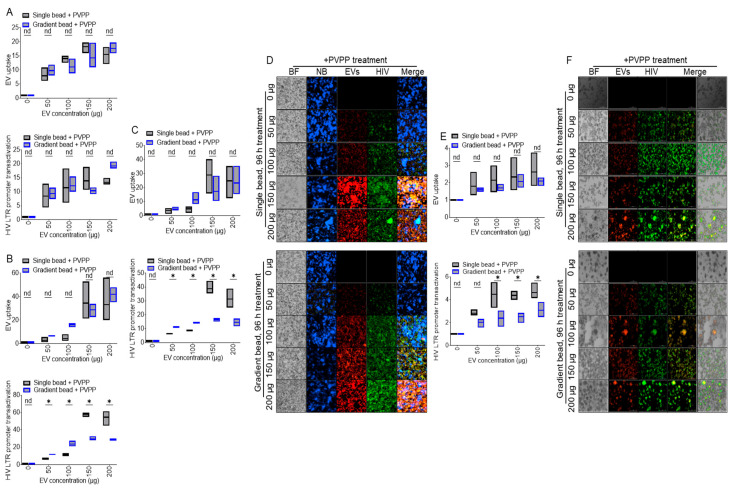
ColEVs isolated with single beads are more effective in transactivating HIV LTR promoter: (**A**) Quantification of JLAT T cells response to ColEVs uptake (top) and level of HIV transactivation (bottom) following 48 h treatment with ColEVs isolated with single or gradient beads in the presence of PVPP. (**B**) Quantification of JLAT T cells response to ColEVs uptake (top) and level of HIV transactivation (bottom) following 72 h treatment with ColEVs isolated with single or gradient beads in the presence of PVPP. (**C**) Quantification of JLAT T cells response to ColEVs uptake (top) and level of HIV transactivation (bottom) following 96 h treatment with ColEVs isolated with single or gradient beads in the presence of PVPP. (**D**) Microscopic images of JLAT T cells response to ColEVs following 96 h treatment with ColEVs isolated with single (top) or gradient (bottom) beads in the presence of PVPP. (**E**) Quantification of TZM-GFP cells response to ColEVs uptake (top) and level of HIV transactivation (bottom) following 96 h treatment with ColEVs isolated with single or gradient beads in the presence of PVPP. (**F**) Microscopic images of TZM-GFP cells response to ColEVs following 96 h treatment with ColEVs isolated with single (top) or gradient (bottom) beads in the presence of PVPP. Statistics were conducted with Prism GraphPad using the Two-stage linear step-up procedure of Benjamini, Krieger and Yekutieli. nd = no difference, *p* * ≤ 0.0001 to 0.0003.

JLAT-TAT-GFP cells were treated with DiR-labeled PBS (0 µg) or various concentrations (50, 100, 150, 200 µg) of ColEVs. Internalization of ColEVs and GFP expression as evidence of HIV LTR promoter reactivation were analyzed at 48 h (Figure 5A, top and bottom), 72 h (Figure 5B, top and bottom), and 96 h (Figure 5C, top and bottom). Visual assessment of uptake of ColEVs (red florescence) and HIV LTR promoter activation (green fluorescence) are shown as representative microscopic images (Figure 5D). The uptake and bioactivity of ColEVs on HIV LTR promoter transactivation was confirmed in TZM-GFP cells at 96 h by quantification of florescence intensities (Figure 5E, top and bottom) and the representative images are shown (Figure 5F). In both JLAT-TAT-GFP and TZM-GFP cells, ColEVs isolated with single beads significantly transactivated HIV LTR promoter at 72 and 96 h post treatment (Figure 5B,C,E, bottom panels).

### 3.8. ColEVs Are Originate from Both Bacteria and Host Particles

**Figure 6:**
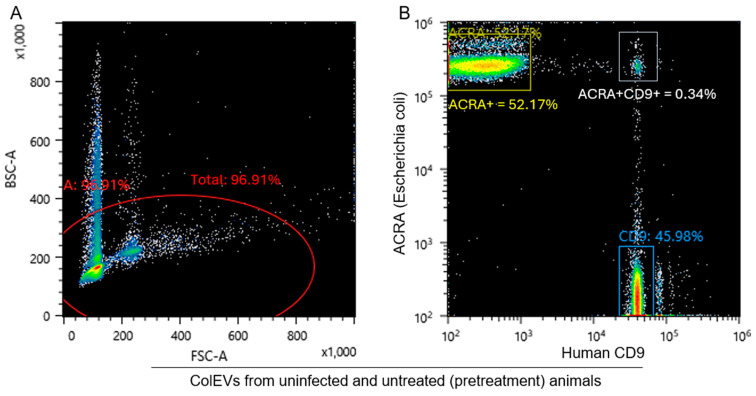
Flow cytometric analysis of the components of ColEVs: (**A**) Gating strategy used to identify CD9+ and AcrA+ ColEVs. (**B**) Plots of AcrA+, CD9+, and AcrA+CD9 ColEVs. Three biological replicates were pooled into one sample for a reliable sorting experiment.

Here, we sought to identify the composition of ColEVs using flow cytometry (Figure 6A). To distinguish ColEVs of bacteria and host origins, we labeled ColEVs with antibodies against host CD9 and *Escherichia coli (E. coli)* AcrA protein (a component of the multi-drug efflux complex AcrAB-TolC that pumps out an extraordinarily wide variety of antibiotics, chemotherapeutic agents, detergents and dyes across two membranes). We found that both CD9+ and AcrA+ ColEVs are present in rhesus macaque GI. 46% of ColEVs are of host origin as they are CD9+, 52% are of bacteria origin as they are AcrA+, while 0.3% of ColEVs may be hybrid particles because they are CD9+AcrA+ (Figure 6B).

## 4. Discussion and Application of the Protocol

ECs|EVs are gaining ground as biomarkers of health, disease, and as therapeutics tools [69,70,71,72], for various diseases, including but not limited to, cancer, neurodegenerative disorders, and infectious diseases. The significance of ECs|EVs demands that rigorous and appropriate isolation methods, such as the use of PPLC are applied when purifying ECs|EVs from various sources, especially from complex biospecimens. The strength of this protocol is that it facilitates the use of the same starting material to separate ECs from EVs and evaluate their cargo composition and their functions. In our prior publication on ECs|EVs, we demonstrated that ECs and EVs have distinct and overlapping cargos [15,16,53,63]. These findings have improved our ability to isolate distinct populations of EPs, determine their composition, and biological functions. While our previous protocols have been focused on body fluid (semen, blood, urine, milk, tissue culture fluids) and tissues (brains), there is a need to develop protocol for isolation of GI-derived ECs|EVs. This present protocol provides a framework for isolation of GI-derived ECs|EVs.

The GI track is a complex environment hosting trillions of microorganisms that may exceed the number of human cells. GI-resident microorganisms secrete molecules that may affect the physiological activities of the host, including metabolism [73], immune response, and disease pathogenesis, such as neurological diseases. Given the ability of ECs|EVs to mediate long and short distant intercellular communication, it is likely that the GI track and liver may crosstalk via ECs|EVs that delivers enteric-derived products, including toxins and GI-microbial-derived ECs|EVs to various organs, such as the liver, heart, brain to reprogram these organs. Thus, GI-derived ECs|EVs may play roles in the detection, pathogenesis, and prevention of diseases, or in the therapy of diseases.

The PPLC modified for GI-derived EPs guarantees a high yield of purified EPs that will be separated into ECs and EVs following pre-separation treatment with PVPP to remove impurities, chromatographic separation using a first-in-class gradient size exclusion column (gSEC), and collection of the different populations of EPs using a fraction collector. The collected analytes are subjected to online ultraviolet-visible (UV–Vis) monitoring of particle spectral profile. The size, yield (in concentration), and surface charge or zeta potential (ζ-potential) of the isolated ECs|EVs are assessed by nanoparticle tracking analysis (NTA), followed by transmission electron microscopy, and Western blotting analysis of EV-specific tetraspanin markers—CD9, CD63, and CD81—cellular uptake, and functional assays.

Before we finalized the efficient protocol described above, we tested different parameters—gradient bead column vs. single bead column, isolation with PVPP vs. isolation without PVPP. Thus, test samples were grouped into four groups: single bead column with PVPP, single bead column without PVPP, gradient bead column with PVPP, and gradient bead column without PVPP. We observed that the EV-rich peaks were more resolved among samples isolated with PVPP inclusive as compared to the samples that were isolated without the addition of PVPP (Figure 2). Additionally, the transmission electron microscopy (TEM) images for samples that were isolated with PVPP were cleaner, with lesser non-EV fragments (Figure 4C), unlike the PVPP-devoid samples (Figure 3C). However, the concentration was higher in samples isolated with gradient beads +PVPP than in samples isolated with single beads + PVPP. EV-specific tetraspanin protein expression (Figure 2B,E) and TEM images (Figure 2G–J) confirmed the abundance of EVs in the isolates in peak 1 of the spectral profile and the absence of ColECs in the analytes. TEM images did not reveal any alteration in the morphology of ColEVs isolated using PVPP. Overall, this study reveals that PPLC guarantees the isolation of a high yield purified EVs from colonic content, and that PVPP further improves the purity of the isolated ColEVs by binding to phenolic compounds and other contaminants that may interfere with the quality and quantity of the isolated products. The isolated ColEVs consist of both bacteria and host particles and are functional since they effectively transactivated HIV LTR promoter. These observations are in line with a previous study where polyvinylpyrrolidone (PVP, a water-soluble polymer) was used for efficient removal of inhibitors that may coprecipitate with DNA during extraction [74].

The use of PVPP for isolating ColEVs may not pose much risk to experimental models (cell lines and animals) because PVPP is authorized as food additive in the European Union (EU) in agreement with Annex II and Annex III to Regulation (EC) No 1333/2008 on food additives [75]. In the USA, PVPP is listed as an approved material in the Adjunct Reference Manual of the Beer Institute and TTB regulations at 27 CFR 24.246.

While the use of PVPP may improve the purity of ColEVs, PVPP may have some unintended consequences. For example, different polyphenolic compounds, including some polyphenol families or sub-families may have specific affinities for PVPP [76]. As a result, PVPP may absorb such polyphenols that may be associated with EVs, resulting in loss of polyphenol-related antioxidant functions responsible for protecting cells against ultraviolet light and disease induced damage, as well as scavenging reactive oxygen species (ROS) in stressed cells [77]. Thus, if PVPP absorbs EV-associated molecules that have potential protective effects, such EVs, may lose their protective effects. It is, thus, advisable to empirically determine the concentration of PVPP to be used based on the specimen and proposed use for the EVs.

Additional studies are required to characterize the proteome, lipidome, RNAome, and functions of ColEVs isolated with and without PVPP to determine the effects of PVPP on the composition and functions of the EVs.

## Data Availability

All data are included within the article and Supplementary Materials: Step-by-step protocol, also available on protocols.io.

## Supplementary Materials

The following supporting information can be downloaded at: https://www.mdpi.com/article/10.3390/cells14080568/s1, Table S1: Bead characteristics; Table S2: Two-way ANOVA—Turkey’s multiple comparisons test. Table S3: Two-way ANOVA—Turkey’s multiple comparisons test. Compare cells with others in its row and its column. References [15,16,52,53,54,69,70,71,72,73] are cited in the Supplementary Materials.
