# Supplementary material for: Rigorous Process for Isolation of Gut-Derived Extracellular Vesicles (EVs) and the Effect on Latent HIV
Source: Cells. 2025 Apr 9;14(8):568. doi: 10.3390/cells14080568 (PMC12025545; doi:10.3390/cells14080568)
Supplement: Supplementary file 1 [file cells-14-00568-s001.zip › cells-3458300-supplementary revised.pdf]

cells

MDPI

Supplementary Materials

Rigorous Process for Isolation of Gut-Derived Extracellular Vesicles and the Effect on Latent HIV

Nneoma C.J. Anyanwu [1], Lakmini S. Premadasa [2], Wasifa Naushad [1], Bryson C. Okeoma [1], Mohan Mahesh [2,*] and Chioma M. Okeoma [1,3*]

[1] Department of Pathology, Microbiology, and Immunology, New York Medical College, Valhalla, NY 10595-1524, USA
[2] Host–Pathogen Interaction Program, Southwest National Primate Research Center, Texas Biomedical Research Institute, San Antonio, TX 78227-5302, USA
[3] Lovelace Biomedical Institute, Albuquerque, NM 87108-5127, USA
* Correspondence: mmohan@txbiomed.org (M.M.); cokeoma@nymc.edu (C.M.O.)

Development and Application of the Protocol

ECs|EVs are gaining ground as biomarkers of health and disease and as therapeutics tools [69–72] for various diseases, including but not limited to cancer, neurodegenerative disorders, and infectious diseases. The significance of ECs|EVs demands that rigorous and appropriate isolation methods, such as the use of PPLC, are applied when purifying ECs|EVs from various sources, especially from complex biospecimens. The strength of this protocol is that it facilitates the use of the same starting material to separate ECs from EVs and evaluate their cargo composition and functions. In our prior publication on ECs|EVs, we demonstrated that ECs and EVs have distinct and overlapping cargos [15,16,53,63]. These findings improved our ability to isolate distinct populations of EPs and determine their composition and biological functions. While our previous protocols focused of bodily fluids (semen, blood, urine, milk, tissue culture fluids) and tissues (brains), there is a need to develop a protocol for the isolation of GI-derived ECs|EVs. This present protocol provides a framework for the isolation of GI-derived ECs|EVs.

The GI track is a complex environment hosting trillions of microorganisms, which may exceed the number of human cells. GI-resident microorganisms secrete molecules that may affect the physiological activities of the host, including metabolism [73], immune response, and disease pathogenesis, such as neurological diseases. Given the ability of ECs|EVs to mediate long- and short-distance intercellular communication, it is likely that the GI track and liver may crosstalk via ECs|EVs to deliver enteric-derived products, including toxins and GI microbial-derived ECs|EVs to various organs, such as the liver, heart, and brain, to reprogram these organs. Thus, GI-derived ECs|EVs may play roles in the detection, pathogenesis, and prevention of diseases or in therapeutic interventions for said diseases.

The PPLC modified for GI-derived EPs guarantees a high yield of purified EPs that will be separated into ECs and EVs following a pre-separation treatment with 1 % PVPP to remove impurities, chromatographic separation using a first-in-class gradient size exclusion column (gSEC), and collection of the different populations of EPs using a fraction collector. The collected analytes are subjected to online ultraviolet–visible (UV–Vis) monitoring of the particle spectral profile. The size, yield (in concentration), and surface charge or zeta potential (ζ-potential) of the isolated ECs|EVs are assessed by nanoparticle tracking analysis (NTA), followed by transmission electron microscopy, Western blotting

 https://doi.org/10.3390/cells14080568

analysis of EV-specific tetraspanin markers—CD9, CD63, and CD81—cellular uptake, and functional assays.

General Laboratory Reagents

1. 1× Dulbecco's phosphate-buffered saline (DPBS, with calcium and magnesium, Corning, Fisher Scientific, Cat. #21-030-CM)
2. 0.1×DPBS (autoclave-sterile)
3. 1 % polyvinylpolypyrrolidone (PVPP)
4. Sephadex beads (G-10, G-15, G-25, G-50, G-75, and G-100)
5. Sample solution (prepared as per experimental requirements)
6. #1 filter paper (Whatman, GE Healthcare, Chicago, IL)
7. 1.5% aqueous uranyl acetate (Electron Microscopy Sciences, Hatfield, PA)
8. NucBlue Live ReadyProbes Reagent (catalog # R37606, Fisher Scientific, Waltham, MA 02451 USA)
9. DiR (1,1-dioctadecyl-3,3,3,3-tetramethylindotricarbocyanine)

General Laboratory Equipment

1. Basix™ 0.2 µm syringe filters, sterile (Fisher Scientific, Cat #13-100-106)
2. 15 mL Falcon tubes
3. 5 mL centrifuge tubes
4. Pipette tips 10 – 1000 µL
5. Pipettes
6. Ultrapure water from a Milli-Q IQ Water Purification System or double-distilled water
7. Genie Vortex Mixer Model: Vortex-Genie 2
8. BioExpress Genemate Rotator W/36X1.5/2ML (with changeable paddles/rotisserie (Cat #490016-746)
9. 4°C refrigerator
10. -80°C freezer
11. Tabletop centrifuge
12. Fractionator (model)
13. Glass columns (25 mL and 100 mL, Bio-Rad)
14. Formvar and carbon-coated 400-mesh copper grids (Electron Microscopy Sciences, Hatfield, PA)
15. Glow discharge unit (e.g., PELCO easiGlow, Ted Pella, Inc., Redding, CA)
16. Parafilm (Bemis Company, Inc., Neenah, WI)
17. Transmission electron microscope (JEM-1400, JEOL, USA, Inc., Peabody, MA)
18. CCD camera (Veleta 2K × 2K, EM-SIS, Germany)
19. 24-well Costar® 24-well Clear TC-treated multiple-well plate (cat. # 3524, Corning, NY 14831 USA)
20. Greiner 96-well plate (cat. #82050-788)
21. Spectrophotometer

Modifications Made to PPLC and Key Steps Involved in the Isolation of Colonic ECs|EVs

1. Soaking of Sephadex beads of various sizes (G-10, G-15, G-25, G-50, G-75, and G-100) in phosphate-buffered saline at 4 °C overnight [16,52,53,54].

2. Packing PPLC 1.0 × 100 cm column with the soaked Sephadex beads layered from smallest to largest [16,52,53,54].

3. Pre-clearing of the colonic contents through flash agitation, rotation, spinning at varying temperatures, and differential centrifugation.

4. Pretreatment of colonic contents with PVPP.

5. Loading of pre-cleared and PVPP-treated and untreated colonic contents onto the PPLC column.

6. Collection of analytes with the fraction collector, spectrophotometric analysis of analyte spectral profile, and storage of analytes.

7. Downstream analysis and characterization of analytes with biochemical and cellular assays.

Preprocessing of Colonic Contents (Thawing and Pre-treatment with PVPP)

The following describes the sample processing or pre-isolation methods after thawing (NB: avoid freeze–thawing samples; all thawed samples should be processed before storing for isolation, if isolation is not possible on the same day).

1. On ice, collect the thawed colon contents with an RNase-/DNase-free sterile semi-microspatula (cat. #CLS3007, ThermoFisher, Pittsburgh, PA 15275, USA) and put in an equal amount of 1× DPBS (1mL/gram) (cat. #MT21030CM, ThermoFisher, Pittsburgh, PA 15275, USA). For example, dissolve 1 mL of colon content in 1 mL of 1× DPBS.

2. At 4 °C, dissolve the mixture by mixing for 30 min using a tube rotator (cat. #9778990, VWR, Wayne, PA 19087, USA) while vortexing (cat. #NC9864336, ThermoFisher, Pittsburgh, PA 15275, USA) intermittently at a high speed for 30 sec every 10 min.

3. Remove insoluble materials and cell debris from the colon content with DPBS and mix by centrifuging the mixture three (3) times at 1600 × g at 4 °C for 5 min. After each centrifugation (cat. #05-400-61, ThermoFisher, Pittsburgh, PA 15275, USA), carefully collect and place supernatants into new tubes before centrifuging again.

4. Repeat centrifugation three (3) times at 5,000 × g at 4 °C for 15 min. Place supernatants into new tubes each time before centrifuging again.

5. Place supernatants in a fresh sterile 15 mL Falcon tube and add 0.1 to 1% polyvinylpolypyrrolidone (PVPP). The PVPP binds to compounds that may interfere with EV isolation and improves the quality of the yield.

6. Vortex for 1 minute and rotate on the tube rotator for 15 min at room temperature.

7. Filter into a new 15 mL Falcon tube with a 0.2 μm syringe filter (cat. #13-100-106, ThermoFisher, Pittsburgh, PA 15275, USA) and centrifuge at 3300 × g for 6 min at 4 °C. This is to remove leftover pelleted PPVP-bound contaminants.

8. Repeat step 7 (NB: Keep samples on ice in-between steps, except when room temperature is required).

9. The resulting colonic biofluid is ready for use for the isolation of EVs by PPLC. Samples can be stored at -80 ℃ if isolation will not be carried out on the same day.

Packing and Equilibration of PPLC Column for Isolation

1. Make 0.1 × DPBS by mixing 1 mL of 1 × DPBS in every 10 mL of double-distilled water (ddH$_2$O). The 0.1 × DPBS is used to soak the beads with which the PPLC columns are packed, equilibrate the column, and rinse the column after isolation.

2. The 100 cm gradient PPLC column should be packed with multi-sized beads as previously described [53]. An empty glass column of 100 cm length, 1 cm inner diameter, and 79 mL volume (Bio-rad cat #7371091) should be packed in-house with a gradient of epichlorohydrin cross-linked dextran beads of various exclusion limits controlled by different degrees of cross-linking. The beads are commercially available from Cytiva and sold under the trade name Sephadex (previously branded for GE Healthcare). The characteristics of the beads are described in the table below. The beads are slowly packed from bottom to top after overnight swelling in 0.1 × DPBS, starting with G-10 and ending with G-100 (**Table S1**).

Table S1. Bead characteristics

**Bead type**	**Catalog number**	**% of column length**[#]	**Particle size distribution range, dry beads (volume share within range %) ***	Exclusion limit (Da)[&]
G-10	17-0010-01	5	40 to 120 (95%)	$< 7 \times 10^2$
G-15	17-0020-01	7.5	40 to 120 (95%)	$< 1.5 \times 10^3$
G-25 fine	17-0032-01	11	20 to 80 (97%)	$1 \times 10^3 – 5 \times 10^3$
G-50 medium	17-0043-01	17.5	50 to 150 (98%)	$1 \times 10^3 – 3 \times 10^4$
G-75	17-0050-01	24	40 to 120 (99%)	$3 \times 10^3 – 8 \times 10^4$
G-100	17-0060-01	35	40 to 120 (98%)	$4 \times 10^3 – 1.5 \times 10^5$

[#] proprietary parameter; * as determined in the certificate of analysis from the manufacturer; and [&] as advertised in the product specifications from the manufacturer at https://www.cytivalifesciences.com/en/us/shop/chromatography/resins/size-exclusion.

3. The column is connected to a low-pressure drop-based small-volume fraction collector (SKU: 171041, Gilson Incorporated, Middleton, WI 53562-0027), which collects the isolated EVs in microplates for UV-Vis spectrometry.

4. With the prepared 0.1 × DPBS, equilibrate the gradient PPLC column with 5 mL of PBS prior to usage. To confirm that the column is ready to use, run the 0.1 × DPBS collected in the microplate on a Biotek Synergy H1 spectrophotometer/microplate reader (Cat #11120531, ThermoFisher, Pittsburgh, PA 15275, USA). A reading less than 1 nm at 230 nm absorbance indicates that the column is well rinsed and ready to use. (Different brands of microplate may give background absorbance. The recommended microplate for use is Greiner, cat. #82050-788, VWR International, Wayne, PA 19087, USA).

Isolation of Colon Content Fluid Via PPLC

1. Place new plates on the stage of the fraction collector.

2. Pipette the colon content fluid onto the gradient column and collect fractions in 96-well with fractionator.

3. Check the absorbance spectra at ranges 280 – 650 nm on the spectrophotometer.

4. Plot a graph of the absorbance peaks (**see Figure 2**) to determine the EV-rich fractions.

5. Pool fractions that are rich in EVs (EV-rich fractions form the first peak on the UV-Vis specter).

6. Store at -80 °C for further downstream application.

Notes

Columns can be reused for as long as the flow rate is optimal. This is usually between 4th and 7th sample isolation.

Ensure that the microplates are washed by soaking for at least 1 hour with 5% bleach, rinsing with absolute ethanol and, finally, double-distilled water before reuse.

Sorting Colonic EVs Using Flow Cytometry

Primary Antibodies:

Biotinylated anti-AcrA
Biotinylated anti-CD9

Bead Information: Size, 5 µm

PolyAn Orange Streptavidin Nanobeads—Ex max 470 – 540 nm, Em max 520 – 580 nm

PolyAn Red4 Streptavidin Nanobeads—Ex max 590 – 680 nm, Em max 660 – 780 nm

There is no spectral overlap of the PolyAn Orange emission (longest wavelength ca. 600 nm) and the PolyAn Red4 emission (shortest wavelength ca. 630 nm), and thus, there is no spectral crosstalk (bleed-through) from the FITC channel into the APC channel using these two dyes.

PolyAn Orange Streptavidin Nanobeads are used for AcrA, and PolyAn Red4 Streptavidin Nanobeads are used for CD9. Treatments include ColEVs being labeled with the antibodies and sorted in two separate tubes, one for each antibody.

Table S2: Two-way ANOVA – Turkey's multiple comparisons test. Compare cell means with others in its row and its column

Number of families = 9; Number of comparisons per row family = 6

Number of comparisons per column family = 10; Alpha = 0.05

Two-way ANOVA – Tukey's multiple comparisons test	Summary	Adjusted P Value
**0 µg ColEVs**		
Single beads -PVPP vs. Gradient beads - PVPP	ns	>0.9999
Single beads -PVPP vs. Single beads +PVPP	ns	>0.9999
Single beads -PVPP vs. Gradient beads +PVPP	ns	>0.9999
Gradient beads -PVPP vs. Single beads +PVPP	ns	>0.9999
Gradient beads -PVPP vs. Gradient beads +PVPP	ns	>0.9999
Single beads +PVPP vs. Gradient beads +PVPP	ns	>0.9999
**50 µg ColEVs**		
Single beads -PVPP vs. Gradient beads -PVPP	ns	0.0612
Single beads -PVPP vs. Single beads +PVPP	*	0.0243
Single beads -PVPP vs. Gradient beads +PVPP	****	<0.0001
Gradient beads -PVPP vs. Single beads +PVPP	ns	0.9801
Gradient beads -PVPP vs. Gradient beads +PVPP	***	0.0008
Single beads +PVPP vs. Gradient beads +PVPP	**	0.0024
**100 µg ColEVs**		
Single beads -PVPP vs. Gradient beads -PVPP	***	0.0004
Single beads -PVPP vs. Single beads +PVPP	****	<0.0001
Single beads -PVPP vs. Gradient beads +PVPP	**	0.0015
Gradient beads -PVPP vs. Single beads +PVPP	****	<0.0001
Gradient beads -PVPP vs. Gradient beads +PVPP	****	<0.0001
Single beads +PVPP vs. Gradient beads +PVPP	ns	0.7723
**150 µg ColEVs**		
Single beads -PVPP vs. Gradient beads -PVPP	*	0.0411
Single beads -PVPP vs. Single beads +PVPP	****	<0.0001

Single beads -PVPP vs. Gradient beads +PVPP	****	<0.0001
Gradient beads -PVPP vs. Single beads +PVPP	****	<0.0001
Gradient beads -PVPP vs. Gradient beads +PVPP	****	<0.0001
Single beads +PVPP vs. Gradient beads +PVPP	***	0.0003
**200 µg ColEVs**		
Single beads -PVPP vs. Gradient beads -PVPP	ns	0.0579
Single beads -PVPP vs. Single beads +PVPP	****	<0.0001
Single beads -PVPP vs. Gradient beads +PVPP	****	<0.0001
Gradient beads -PVPP vs. Single beads +PVPP	****	<0.0001
Gradient beads -PVPP vs. Gradient beads +PVPP	****	<0.0001
Single beads +PVPP vs. Gradient beads +PVPP	ns	0.8664

Table S3: Two-way ANOVA – Turkey's multiple comparisons test. Compare cell means with others in its row and its column

Number of families = 9; Number of comparisons per row family = 6

Number of comparisons per column family = 10; Alpha = 0.05

Two-way ANOVA – Tukey's multiple comparisons test	Summary	Adjusted P Value
**0 µg ColEVs**		
Single beads -PVPP vs. Gradient beads -PVPP	ns	>0.9999
Single beads -PVPP vs. Single beads +PVPP	ns	>0.9999
Single beads -PVPP vs. Gradient beads +PVPP	ns	0.9978
Gradient beads -PVPP vs. Single beads +PVPP	ns	>0.9999
Gradient beads -PVPP vs. Gradient beads +PVPP	ns	0.9977
Single beads +PVPP vs. Gradient beads +PVPP	ns	0.9994
**50 µg ColEVs**		
Single beads -PVPP vs. Gradient beads -PVPP	ns	0.8759
Single beads -PVPP vs. Single beads +PVPP	****	<0.0001
Single beads -PVPP vs. Gradient beads +PVPP	****	<0.0001
Gradient beads -PVPP vs. Single beads +PVPP	****	<0.0001
Gradient beads -PVPP vs. Gradient beads +PVPP	****	<0.0001
Single beads +PVPP vs. Gradient beads +PVPP	ns	>0.9999
**100 µg ColEVs**		
Single beads -PVPP vs. Gradient beads -PVPP	ns	0.1766
Single beads -PVPP vs. Single beads +PVPP	****	<0.0001
Single beads -PVPP vs. Gradient beads +PVPP	****	<0.0001
Gradient beads -PVPP vs. Single beads +PVPP	****	<0.0001
Gradient beads -PVPP vs. Gradient beads +PVPP	****	<0.0001
Single beads +PVPP vs. Gradient beads +PVPP	ns	0.9992
**150 µg ColEVs**		
Single beads -PVPP vs. Gradient beads -PVPP	ns	0.3410
Single beads -PVPP vs. Single beads +PVPP	****	<0.0001
Single beads -PVPP vs. Gradient beads +PVPP	****	<0.0001
Gradient beads -PVPP vs. Single beads +PVPP	****	<0.0001
Gradient beads -PVPP vs. Gradient beads +PVPP	****	<0.0001
Single beads +PVPP vs. Gradient beads +PVPP	ns	0.9995
**200 µg ColEVs**		
Single beads -PVPP vs. Gradient beads -PVPP	ns	0.1318
Single beads -PVPP vs. Single beads +PVPP	****	<0.0001
Single beads -PVPP vs. Gradient beads +PVPP	****	<0.0001
Gradient beads -PVPP vs. Single beads +PVPP	****	<0.0001
Gradient beads -PVPP vs. Gradient beads +PVPP	****	<0.0001
Single beads +PVPP vs. Gradient beads +PVPP	ns	0.9852

ns = not significant, **** $p \leq 0.0001$.
